# Effects on Serum Protein Levels From One Bout of High Intensity Interval Training in Individuals With Axial Spondyloarthritis and Controls

**DOI:** 10.1002/iid3.70305

**Published:** 2025-12-14

**Authors:** Åsa Andersson, M. Charlotte Olsson, Anna Torell, Elisabeth Mogard, Emma Haglund

**Affiliations:** ^1^ Department of Environmental‐ and Bioscience Halmstad University Halmstad Sweden; ^2^ Department of Rehabilitation Ängelholm Hospital Ängelholm Sweden; ^3^ Rheumatology Lund University and Skåne University Hospital Lund Sweden; ^4^ Spenshult Research and Development Center Halmstad Sweden

**Keywords:** axial spondyloarthritis, BMP‐7, CRP, exercise rehabilitation, high‐intensity interval training, IL‐6

## Abstract

**Background:**

Axial spondyloarthritis (axSpA) is a chronic inflammatory disease primarily affecting the axial skeleton causing pain, inflammation, and stiffness. Individuals with axSpA are at greater risk of developing cardiovascular disease, which can be counteracted by physical activity. High‐intensity interval training (HIIT) has been shown to improve cardiovascular health, but the effect on disease activity and the level of inflammation in axSpA has been less studied. In the herein presented pilot study, the aim was to investigate if serum levels of inflammatory cytokines, myokines, and protein markers for bone metabolism are acutely affected by one bout of HIIT in individuals with axSpA and healthy controls (HC).

**Methods:**

Ten participants with axSpA (medication: 6 NSAIDs, 3 biologics, 1 no‐treatment) and 11 age‐ and sex‐matched HC performed a single HIIT bout on a cycle ergometer: 4 × 4 min intervals with 3 min active rest in between. Blood samples were taken before and 1 h after the HIIT bout. Serum proteins (IL‐6, IL‐17, IL‐18, TNF‐α, CXCL‐10, VEGF‐A, BDNF, DKK‐1, osteoprotegerin, osteocalcin, osteopontin, BMP‐7, CRP) were analyzed with a Luminex system or ELISA. A two‐way ANOVA was used for comparisons.

**Results:**

A main effect from baseline to 1 h post HIIT showed that both groups had a significant increase in serum levels of IL‐6. VEGF‐A was significantly lower in the axSpA group but was not affected by the HIIT bout. BMP‐7 increased in both groups after the HIIT. For the other proteins analyzed, there were no significant differences in serum concentrations between individuals with axSpA and HC, or within the two groups before and after one bout of HIIT.

**Conclusions:**

One acute bout of HIIT significantly increases the serum concentrations of IL‐6 and BMP‐7 after 1 h in both individuals with axSpA and HC, whereas serum levels of other proteins investigated are not changed.

**Abbreviations**
axSpA: axial spondyloarthritis
BDNF: brain derived neurotrophic factor
BMP‐7: bone morphogenetic protein‐7
CRP: C‐reactive protein
DKK‐1: Dickkopf‐related protein 1
HC: healthy controls
HIIT: high‐intensity interval training
IL‐6, ‐17, ‐18: interleukin‐6, ‐17, ‐18
NSAID: nonsteroidal anti‐inflammatory drugs
OC: osteocalcin
OPG: osteoprotegerin
OPN: osteopontin
TNFa: tumor necrosis factor alpha
VEGF: vascular endothelial growth factor

## 1 Background

Spondyloarthritis is an umbrella term for a group of inflammatory rheumatic diseases, including axial spondyloarthritis (axSpA), which primarily affects the axial skeleton and the sacroiliac (SI)‐joint [1]. The clinical manifestations encompass stiffness, aching, and pain around the spine and pelvis and the disease process can lead to fusing of the SI‐joint, but also of the vertebrae (i.e., ankylosis) [2, 3, 4]. AxSpA includes patients with structural changes on X‐ray in the SI‐joint or spine, in addition to patients without X‐ray verified changes (non‐radiographic axSpA) [1].

Studies have shown that individuals with axSpA have an increased risk of cardiovascular disease, which influences the treatment of the disease and overall health [5]. The elevated cardiovascular risk is believed to be the result of a chronic systemic inflammation and insufficient level of physical activity, but other comorbidities might play a role as well [6, 7]. Recommended treatment of axSpA is a combination of pharmacological and nonpharmacological treatment including exercise interventions [8, 9], but to date, the most suitable form of exercise for individuals with axSpA is not known [8, 10]. Exercise guidelines for individuals with axSpA include domains of cardiovascular‐, flexibility‐, and resistance training. Moreover, guidelines need to be individually tailored and be based on shared decision making [8, 10].

Traditionally, exercise interventions for individuals with axSpA involve moderate intensity exercise training, but lately some researchers have promoted the incorporation of high intensity interval training (HIIT) [11, 12]. HIIT is described as high intensity exercise bouts separated by periods of active recovery phases. It has been demonstrated that regular HIIT bouts can lead to better cardiovascular health through increased VO_2max_ and improved muscle metabolism [13, 14]. Moreover, studies in metabolic disorders show that HIIT has a positive effect on systemic inflammatory markers linked to the disease state [15, 16]. The significance of HIIT for disease development in rheumatic disorders in general, including the response in levels of inflammatory markers, have not been widely studied. A 3‐month HIIT intervention improved disease symptoms and cardiovascular health [11, 12]. Levels of pro‐inflammatory cytokines were not affected when measured 48 h after the HIIT‐bout [11]. During extensive muscle activity, a large set of proteins, called myokines, are released which are suggested to influence many organ systems with impact on metabolism and inflammation among other functions [17, 18]. One of the most studied myokines, IL‐6, is immediately released in response to muscle activity [19]. IL‐6 is a pleiotropic cytokine with both pro‐inflammatory and anti‐inflammatory effects. The release of myokines, like IL‐6, upon exercise is suggested to counteract inflammation and, thus, cardiovascular issues in rheumatic diseases, since systemic inflammation is an important factor in atherosclerosis [18, 20]. Other examples of proteins produced by muscle and other tissues are brain derived neurotrophic factor (BDNF), a neurotrophin with effects on synapse activities in the brain [21], and vascular endothelial growth factor (VEGF) important for vascularization of skeletal muscle tissue in response to exercise training [22].

The ankylosis that features axSpA is part of bone metabolism, which extends from the formation of new bone (syndesmophytes) to osteoporosis and bone erosion [23]. It is not yet fully understood whether bone metabolism with new bone formation is linked to the inflammatory process in axSpA. Moreover, changes in levels of proteins involved in bone metabolism in response to exercise training have not been studied in this disease.

To safely recommend HIIT training to individuals with axSpA, it is important to first study the acute effects after a single HIIT bout regarding disease symptoms and state of inflammation. Previous studies on the acute effect of a single bout of HIIT in healthy adults, overweight, and obese individuals have shown that changes in levels of serum inflammatory markers are dependent on exercise intensity and metabolic status [24], and that there is no severe inflammation due to muscle damage [25].

With an overall research aim to study the role for HIIT as an exercise intervention in individuals with axSpA regarding influence on systemic inflammation and bone metabolism, in addition to the effect on lowering of the risk for cardiovascular disease, we performed the herein presented study. This cross‐sectional pilot study was undertaken to provide a controlled evaluation of any immediate effects of one HIIT bout on disease activity measured through serum levels of a set of inflammatory cytokines, myokines, and markers for bone metabolism in age‐ and sex‐matched individuals with axSpA and HC.

## 2 Methods

### 2.1 Sample Size Calculation

The sample size for a 2*2 ANOVA design was calculated (G*power) based on previous results for IL‐6 after an acute bout of HIIT in healthy individuals [26, 27]. At a significance level of 5%, a confidence level of 95%, and an effect size of 0.4, the analysis indicated that a sample size of at least 24 participants, with 12 in each group, was needed for the study. After inclusion, the study consisted of 21 individuals (10 with axSpA and 11 age and sex‐matched HC).

### 2.2 Participants

The study included 21 individuals (10 females) between the ages of 18 and 50 (mean ± SD age 40 ± 7). 10 participants from two rheumatology clinics in southern Sweden were diagnosed with axSpA according to the Assessment in SpondyloArthritis international Society (ASAS) classification criteria [28] and 11 participants were age‐ and sex‐matched HC. Participants were included irrespective of their level of disease activity. Exclusion criteria were cardiovascular disease, lung disease, or any other disease or symptom that reduced the physical capacity or the possibility of completing a HIIT bout (e.g. high blood glucose, diabetes, hypertension, lung diseases, or symptoms of angina, chest pain, dizziness or severe shortness of breath while exercising*)*. Those with changed medication in the previous 3 months, deviating electrocardiogram, and/or pregnancy were also excluded. 8 of 10 individuals in the axSpA group and 7 of 11 in the HC group reported that they performed physical exercise more than 60 min per week. The individuals in the axSpA group, except for one participant, used anti‐inflammatory drugs such as Nonsteroidal anti‐inflammatory drugs (NSAIDs, *n* = 3) and biologics (*n* = 6). Participants were asked to avoid food and drinks 2 h before arrival, tobacco 30 min before, and to avoid exercising at high intensity the evening before the testing procedures (see below) and the HIIT bout. The study was approved by the national ethical review board (Dnr: 2019‐04155 and Dnr: 2022‐03114‐02) and followed the ethical principles of the declaration of Helsinki.

### 2.3 Testing Procedures

Before inclusion, electrocardiogram (ECG) and blood pressure were measured. In the event of deviating results regarding ECG and/or a blood pressure > 140/90, the person was excluded and referred to the health center for further investigation. Anthropometrics such as height was measured with a stadiometer and body composition with bioelectrical impedance analysis (InBody 770, Body Space South Korea) (Table 1). Thereafter, participants were fitted with a heart rate (HR) monitor (Polar, Finland) and the Åstrand's submaximal cycle ergometer (Monark, Vansbro, Sweden) test for prediction of maximal oxygen consumption (VO_2max_) was performed [29].

**Table 1:** Baseline data for participants with axial spondyloarthritis (axSpA) and age‐ and sex matched healthy controls (HC).

	axSpA (*n* = 10)	HC (*n* = 11)	*p* value^a^
Age	41 (7)	40 (7)	0.705
Sex (men/women)	5/5	5/6	NA
Skeletal muscle mass (kg)	32.8 ± 5.8	33.5 ± 8.5	0.705
Body fat (%)	22.0 ± 5.8	19.8 ± 7.8	0.468
Systolic blood pressure (mm Hg)	127 (11)	128 (9)	0.710
Diastolic blood pressure (mm Hg)	81 (6)	80 (5)	0.824
VO_2_max (ml O_2_/kg/min)	39.9 ± 8.4	42.6 ± 10.7)	0.973
Smoker or using tobacco in other forms	3	2	NA
EQ‐5D, 0–1^b^ health status	0.87 (0.11)	0.93 (0.10)	0.251
BASDAI, 0–10^c^ disease activity	1.6 (0.8)	NA	NA
BASFI, 0–10^c^ physical function	0.7 (0.8)	NA	NA
BASG 0–10^c^ wellbeing	1.5 (1.0)	NA	NA
Anti‐inflammatory drugs^d^	9/10	2/11	NA
Drugs other than anti‐inflammatory^e^	0/10	1/11	NA

*Note:* Results presented as mean (±SD). BASDAI, Bath Ankylosing Spondylitis Disease Activity Index; BASFI, Bath Ankylosing Spondylitis Functional Index; BASG, Bath Ankylosing Spondylitis Global.

^a^ Difference between axSpA and HC analyzed with Mann‐Whitney *U*‐test.

^b^ Worst to best.

^c^ Best to worst.

^d^ Nonsteroidal anti‐inflammatory drugs (NSAID) (axSpA *n* = 3), TNF‐α‐inhibitors (axSpA *n* = 6), Corticosteroids (allergy/asthma) (HC *n* = 2).

^e^ Synthetic thyroid hormone (HC *n* = 1).

After a brief rest, participants began the HIIT‐bout while wearing a HR monitor. HIIT was performed on a rpm‐independent ergometer bike (LC‐6 or 928E, Monark, Vansbro. Sweden) and included four high intensity intervals, each 4 min in duration, with 3 min active rest (about 70% of maximum HR) between intervals. During the high intensity intervals, the participants should reach > 17 on the rating of the perceived exertion (RPE) scale [30] and reach 90% of estimated maximum HR (HR_max_) any time during the interval [31]. After the HIIT‐bout, the participants filled out the disease‐specific questionnaire Bath Ankylosing Spondylitis Disease Activity Index (BASDAI) on disease activity scoring 0–10 (best‐worst) [32]. For descriptive data, health status was measured with the generic questionnaire EuroQol‐5 domain (EQ‐5D) consisting of five questions covering mobility, self‐care, usual activity, pain/discomfort, and anxiety/depression scoring 0‐1 (no health to full health) [33].

Blood samples were collected before (baseline) and 1 h after the HIIT‐bout ended. Blood samples rested for 30 min followed by centrifugation for 10 min. The serum supernatant was aliquoted and stored at ‐80°C in biobank number 85 at FoU Spenshult, Halmstad. Baseline plasma levels of lipids (P‐cholesterol, P‐triglycerides, P‐LDL, P‐HDL) and glucose were analyzed at clinical chemistry departments at the nearest hospitals.

### 2.4 Serum Analyzes of Cytokines, Myokines, and Bone‐Associated Proteins

Serum was analyzed with the Luminex MAGPIX (Luminex corporation, Austin, US) and ELISA (Spectramax Plus 384, Molecular Devices, San Jose, US). Selected markers from the Human Cytokine/Chemokine/Growth Factor Panel A (HCYTA‐60K), Human Myokine Panel (HMYOMAG‐56K), and Human Bone Panel (HBNMAG‐51K) (Merck KGaA, Darmstadt, Germany) were used for the analysis of IL‐6, IL‐17, IL‐18, TNFa, CXCL‐10, VEGF‐A, BDNF, DKK‐1, osteoprotegerin, osteocalcin, and osteopontin. The Belysa Immunoassay Curve Fitting Software (Merck KGaA, Darmstadt, Germany) was used for analysis of Luminex MAGPIX data. CRP and Bone Morphogenetic Protein (BMP‐7) were analyzed with Human C‐reactive protein and BMP‐7 Quantikine ELISA (R&D systems INC Minneapolis, US), respectively, according to the manufacturers protocol. For ELISA data, the “Quest Grap Four Parameter Logistic (4PL) Curve Calculator.” *AAT Bioquest, Inc*. https://www.aatbio.com/tools/four-parameter-logistic-4pl-curve-regression-online-calculator was used for the standard curve fitting. The lowest limit of detection for the different serum proteins was: IL‐6 (0.18 pg/ml), IL‐17 (0.89 pg/ml), IL‐18 (0.42 pg/ml), CXCL‐10 (2.47 pg/ml), TNF‐α (4.4 pg/ml), VEGF‐A (0.91 pg/ml), BDNF (3 pg/ml), DKK‐1 (1.2 pg/ml), osteoprotegerin (1.8 pg/ml), osteocalcin (37.5 pg/ml), osteopontin (15.6 pg/ml), BMP‐7 (2.44 pg/ml), CRP (0.010 ng/ml). For a few samples some protein levels were below detection as indicated in Table 2.

**Table 2:** Serum protein levels in individuals with axial spondyloarthritis (axSpA) and age‐ and sex matched healthy controls (HC) at baseline and 1 h after a 4 min x four times high intensity interval training (HIIT) session.

Serum analyte	axSpA baseline (*n* = 10)	HC baseline (*n* = 11)	axSpA post‐HIIT (*n* = 10)	HC post‐HIIT (*n* = 11)	2*2 ANOVA *p*‐value	Post‐Hoc *p* value
IL‐6 (pg/ml)	2.2 ± 3.0	0.4 ± 0.4	3.2 ± 1.8	1.9 ± 2.0	MEpre‐post	AxSpA 0.23
	*n* = 8^a^	*n* = 10	*n* = 8	*n* = 10	0.03	HC 0.04
IL‐17 (pg/ml)	3.5 ± 5.1	4.9 ± 3.1	3.7 ± 4.5	4.8 ± 3.2	> 0.05	NA
	*n* = 5	*n* = 8	*n* = 5	*n* = 8		
IL‐18 (pg/ml)	14.9 ± 7.1	24.4 ± 14.4	16.2 ± 6.8	24.7 ± 14.6	> 0.05	NA
	*n* = 9	*n* = 10	*n* = 9	*n* = 10		
TNFα (pg/ml)	51.6 ± 94.0	29.0 ± 47.9	48.2 ± 75.1	41.2 ± 88.2	> 0.05	NA
CXCL‐10 (pg/ml)	82.7 ± 65.1	68.9 ± 35.4	75.7 ± 54.5	66.3 ± 31.4	> 0.05	NA
VEGF‐A (pg/ml)	159 ± 138	326 ± 184	172 ± 122	325 ± 170	ME group	Baseline 0.031
					0.03	Post 0.030
BDNF (ng/ml)	15.6 ± 5.2	15.8 ± 3.9	16.1 ± 3.9	16.3 ± 3.3	> 0.05	NA
DKK‐1 (pg/ml)	1744 ± 379	1527 ± 212	1767 ± 364	1549 ± 207	> 0.05	NA
OPG (pg/ml)	280 ± 68	261 ± 39	278 ± 57	267 ± 47	> 0.05	NA
OC (ng/ml)	16.2 ± 9.1	14.5 ± 5.3	16.1 ± 8.4	15.1 ± 6.0	> 0.05	NA
		*n* = 10		*n* = 10		
OPN (ng/ml)	18.4 ± 14.3	17.8 ± 6.8	14.8 ± 11.1	16.7 ± 9.1	> 0.05	NA
BMP‐7 (pg/ml)	61.6 ± 13.1	64.6 ± 20.8	75.2 ± 20.0	75.0 ± 17.8	ME pre‐post < 0.001	AxSpA 0.004
						HC 0.018
CRP (µg/ml)	1.12 ± 1.06	0.77 ± 0.85	1.11 ± 1.12	0.71 ± 0.75	> 0.05	NA

*Note:* All values are means ± standard deviations.

^a^ Number of values included in the calculation if different from the total number; ME, main effect; IL‐6,‐17,‐18, Interleukin‐6,‐17,‐18; TNF‐α, Tumor Necrosis Factor alpha; CXCL‐10, C‐X‐C motif chemokine ligand 10; VEGF‐A, Vascular Endothelial Growth Factor‐A; BDNF, Brain Derived Neurotrophic Factor; DKK‐1, Dickkopf WNT signaling pathway inhibitor 1, OPG, Osteoprotegerin; OC, Osteocalcin; OPN, Osteopontin; BMP‐7, Bone Morphogenetic Protein‐7; CRP, C‐reactive protein.

### 2.5 Statistics

Statistical analyzes were performed with SPSS (version 28.0, IBM SPSS Statistics, IBM corp. USA). Differences in baseline descriptive data was calculated with Mann‐Whitney *U* test. A repeated measures analysis of variance (ANOVA) was used to detect differences between groups and before and after a single HIIT bout in a 2 (group) * 2 (pre‐post) design. For main effects or interactions significant at *p* ≤ 0.05, Least Significant Difference (LSD) post‐hoc analyzes were performed. Data is reported as mean ± standard deviation (SD) and statistical significance was set to *p* ≤ 0.05.

## 3 Results

Twenty‐two participants were recruited, of which one was excluded due to abnormal ECG in the clinical screening. Ten individuals diagnosed with axSpA (age 41 ± 7 years), and 11 HC (mean age 40 ± 7 years) completed the HIIT‐bout. Physiological measurements, values of self‐reported disease activity and health status, are shown in Table 1. Except for the self‐reported disease parameters not applicable for HC, the participants in the two groups were well matched for body composition (skeletal muscle mass: axSpA 32.8 ± 5.8, HC 33.5 ± 8.5 kg; body fat: axSpA 22.0 ± 5.8, HC 19.8 ± 7.8%), resting blood pressure (axSpA 127/81 ± 11/6, HC 128/80 ± 9/5 mmHg) and maximal oxygen consumption (VO_2max_) (axSpA 40 ± 8, HC 43 ± 10 mlO_2_/kg/min) (Table 1). Furthermore, no significant differences were observed for CRP (Table 2), plasma lipoproteins, triglycerides, and glucose (data not shown). All participants with axSpA, except one, were treated with anti‐inflammatory drugs (Table 1).

### 3.1 Baseline and Post‐HIIT Levels of Cytokines, Myokines and Bone‐Associated Proteins

Sera from the two participating groups were analyzed for proteins involved in inflammation and bone metabolism, in addition to myokines (Table 2). A significant ANOVA pre‐post main effect (*p* = 0.03) was seen for IL‐6 (pg/ml): axSpA 2.2 (3.0) to 3.2 (1.8) and HC 0.4 (0.4) to 1.9 (2.0). Post‐hoc analyzes showed increased levels of IL‐6 in HC from baseline to post‐HIIT (*p* = 0.04) but not in axSpA (*p* = 0.23). There was no difference in the level of IL‐6 (pg/ml) between the axSpA and HC groups at baseline: axSpA 2.2 (3.0) and HC 0.4 (0.4) (*p* = 0.07) or post‐HIIT: axSpA 3.2 (1.8) and HC 1.9 (2.0) (*p* = 0.20) (Table 2). We found a significant group main effect (*p* = 0.03) for the level of serum VEGF‐A (pg/ml), with lower levels in the axSpA group at both baseline: axSpA 159 (138) and HC 326 (184) (*p* = 0.03), and post‐HIIT: axSpA 172 (122) and HC 325 (170) (*p* = 0.03) (Table 2). No differences in serum levels (ANOVA *p* > 0.05) for any of the remaining myokines and cytokines analyzed were observed between or within the axSpA and HC groups at baseline and post‐HIIT (Table 2). For the bone associated proteins a significant pre‐post main effect (*p* < 0.001) was found for BMP‐7. The serum‐concentration of BMP‐7 (pg/ml) increased significantly from baseline: axSpA 61.6 (13.1) and HC 64.6 (20.8), to post‐HIIT: axSpA 75.2 (20) (*p* = 0.004) and HC 75.0 (17.8) (*p* = 0.02) (Table 2).

## 4 Discussion

HIIT is an exercise form that has become popular in recent years, not only in healthy populations but also in individuals with different chronic diseases. Briefly, the health benefits associated with HIIT are: increased cardiovascular capacity, lower blood pressure and improved arterial function [34, 35, 36]; increased metabolic health with weight control and improved insulin sensitivity [37]; increased muscle strength; and influence on mental health with reports on reduced depression and anxiety [38]. HIIT has, however, not been extensively studied as an exercise intervention in individuals with various rheumatic disorders, and therefore it is important to understand the acute effects of a HIIT‐bout on disease symptoms and the state of inflammation before starting a HIIT‐intervention. In the present pilot study, we investigated a set of serum markers and physiological measurements in age‐ and sex‐matched individuals with axSpA and HC before and after one bout of HIIT with the aim of determining levels of specific serum markers in axSpA and how these are affected by one acute bout of HIIT. One goal with the present and other ongoing studies in our group, is to establish biomarkers that could be used for the evaluation of disease progression and the effect of regular HIIT bouts on the disease process to optimize and individualize HIIT‐based interventions. Furthermore, due to the systemic inflammation, individuals with axSpA are at higher risk for developing cardiovascular disease and HIIT has been shown to be a possible intervention to manage the disease and comorbidities when appropriate [12].

In this study, we analyzed serum markers including proinflammatory cytokines, myokines, and proteins involved in bone metabolism, in addition to CRP (Table 2). Except for IL‐6, no significant differences in cytokine levels were observed between the two groups at baseline or post‐HIIT. The acute increase in IL‐6 levels, where the levels from baseline to post‐HIIT increased more in the HC than in axSpA, is consistent with previous studies on the effect of one acute bout of HIIT on serum IL‐6 [27, 39, 40]. We choose to measure the serum analytes 1 h after the HIIT bout, since this time point may detect proteins released already during the HIIT bout, but also proteins that emerge in serum at different times after the HIIT bout. In a preliminary study on 12 healthy young individuals (age 18–29), we showed that already at 5 min post‐HIIT, the levels of IL‐6 increased 2.4 times compared to the baseline level. After 1 h this increase was only 1.6 times the baseline level (see Additional file 1). This is in line with previous studies [27, 40] and indicates that measuring the IL‐6 serum concentration already at 5 min post HIIT may show a more exact increase in the individual production of IL‐6.

The pathogenesis of axSpA is strongly dependent on the genetic linkage to HLA‐B‐27, but other associated genes have also been identified [1]. Nongenetic factors suggested to be involved in the triggering of the disease are gut dysbiosis and mechanical stress contributing to enthesitis and pathology in the subchondral bone [2]. It is believed that inflammation is primarily driving bone destruction, but there might also be an interaction between inflammation‐driven factors and new bone formation ongoing in the inflamed tissue. We did not find any differences in the levels of analyzed proteins involved in bone metabolism (DKK‐1, OPG, OC, OPN), except for BMP‐7 where an increase in serum concentration after HIIT was found in both groups (Table 2). The bone morphogenetic proteins (BMPs) belong to the transforming growth factor (TGF)‐ß family and have important roles in osteogenesis and bone formation [41]. It has been suggested that new bone formation in axSpA is partly driven by BMPs [42]. BMP‐7 is a growth factor involved in many cellular processes, including those having anti‐inflammatory effects [41, 43], but is considered most important for bone formation [44]. Studies have shown an increase in levels of BMP‐7 in axSpA patients [45] with a correlation to radiographic structural damage, which is linked to new bone formation [46, 47]. To our knowledge, BMP‐7 has not been studied in the context of HIIT, but it has been shown that BMP‐7 levels decrease with increasing age and that exercise and subsequent release of myokines, including BMP‐7, is important for bone health in the elderly [44]. The fact that we observed an increase in serum concentrations of BMP‐7 post HIIT, may indicate that HIIT can contribute to improvement of bone metabolism as well as inflammation through BMP‐7. More research is however needed to understand the role for BMP‐7 in axSpA with regard to pathological bone formation versus anti‐inflammatory features.

VEGF‐A is a main signaling factor in angiogenesis by regulating proliferation of endothelial cells and vascular permeability. It is released by many different cell types and can activate inflammatory cells and the production of proinflammatory mediators. The level of VEGF in healthy adults has been shown not to be influenced by HIIT in the short term, which is in line with the present results [48, 49]. In individuals with axSpA VEGF‐levels are elevated, but the levels are significantly decreased by anti‐inflammatory treatment such as TNFa inhibitors [50, 51]. The participants with axSpA in the present study had lower levels of VEGF‐A compared to the HCs, which could be explained by the fact that all, but one, were on treatment with anti‐inflammatory drugs. Similarly, there was no difference in the baseline levels of CRP between the two groups. Reports from studies on the effect of HIIT on CRP levels have shown conflicting results probably due to a variation in disease status and group size [38, 52]. In the present study there was no significant difference in CRP levels between the axSpA and HC groups and we did not observe any changes in CRP levels from baseline to post‐HIIT. Taken together, the results suggest that one bout of HIIT does not change the level of inflammatory markers in axSpA patients and HC.

The present study was performed to address the impact of one single bout of HIIT on serum levels of inflammatory cytokines, myokines, and markers for bone metabolism in individuals with axSpA and HC. One limitation of the study is the small group sizes, which however were well matched regarding sex, age, and physiological measures. Due to the small groups, there was not sufficient power in the calculations to analyze sex differences for the different serum markers. In addition, all participants with axSpA, except one, were treated with anti‐inflammatory drugs, which had a considerable impact on certain serum markers. It is, however, difficult to avoid the effects of anti‐inflammatory treatment as it is not ethically justifiable to withdraw medication before a study. Another limitation is that participants with high disease activity cannot be included. According to national treatment guidelines for axSpA in Sweden, patients with a BASDAI score > 4 are recommended to start or, if they have already started, switch pharmacological treatment. Consequently, it was not possible to recruit individuals with persistent high disease activity who were on stable medication, which may have affected the generalizability of the study results.

Further studies should aim to identify stable biomarkers to determine the long‐term dose–response of exercise programs in individual axSpA patients to reduce or halt the chronic inflammatory process and its effect on bone metabolism and cardiovascular disease. Biomarkers that are less affected by anti‐inflammatory pharmacological treatment would be preferable for evaluating the actual disease status in combination with self‐reported disease data, and how exercise of different types, intensities, and durations affects the disease process.

## 5 Conclusion

In this study we investigated how one bout of HIIT acutely affect serum levels of specific pro‐ and anti‐inflammatory cytokines, myokines, and proteins involved in bone metabolism in age‐ and sex‐matched individuals with axSpA compared to HC. From the herein presented results we conclude that one bout of HIIT significantly increases the serum concentrations of IL‐6 and the bone associated protein BMP‐7 in both groups, but that the concentrations of none of the other proteins investigated, including CRP and VEGF‐A, were significantly changed. The result indicates that one acute bout of HIIT is safe and feasible and has no significant impact on the inflammatory status in individuals with axSpA. To optimize future exercise interventions for this population, further research is needed to evaluate the long‐term effects of HIIT on biomarkers and self‐reported disease outcomes.

## Author Contributions

**Åsa Andersson:** conceptualization, data curation, formal analysis, funding acquisition, investigation, methodology, project administration, validation, writing – original draft, writing – review and editing. **M. Charlotte Olsson:** conceptualization, data curation, formal analysis, funding acquisition, investigation, methodology, project administration, validation, writing – original draft, writing – review and editing. **Anna Torell:** investigation, writing – review and editing. **Elisabeth Mogard:** investigation, writing – review and editing. **Emma Haglund:** conceptualization, data curation, formal analysis, funding acquisition, investigation, methodology, project administration, validation, writing – original draft, writing – review and editing.

## Ethics Statement

The study was approved by the Swedish Ethics Review Authority (Dnr: 2019‐04155 and Dnr: 2022‐03114‐02) and performed in accordance with the Declaration of Helsinki. Each participant signed an informed consent for participation in the study.

## Consent

The authors have nothing to report.

## Conflicts of Interest

The authors declare no conflicts of interest.

## Supporting information


**Supplementary Table 1:** Fold change of IL‐6 in serum of healthy individuals (age 18–29) from baseline and from 5 min post HIIT on a group level.

## Data Availability

The data sets used and/or analyzed during the current study are available from the corresponding author on reasonable request.

## References

[1] J. Sieper and D. Poddubnyy , “Axial Spondyloarthritis,” Lancet 390, no. 10089 (2017): 73–84. 28110981 10.1016/S0140-6736(16)31591-4

[2] V. Navarro‐Compán , A. Sepriano , B. El‐Zorkany , and D. van der Heijde , “Axial Spondyloarthritis,” Annals of the Rheumatic Diseases 80, no. 12 (2021): 1511–1521. 34615639 10.1136/annrheumdis-2021-221035

[3] J. Sieper and D. van der Heijde , “Review: Nonradiographic Axial Spondyloarthritis: New Definition of an Old Disease?,” Arthritis & Rheumatism 65, no. 3 (2013): 543–551. 23233285 10.1002/art.37803

[4] J. A. Walsh and M. Magrey , “Clinical Manifestations and Diagnosis of Axial Spondyloarthritis,” JCR: Journal of Clinical Rheumatology 27, no. 8 (2021): e547–e560. 33105312 10.1097/RHU.0000000000001575 PMC8612900

[5] E. Toussirot , “The Risk of Cardiovascular Diseases in Axial Spondyloarthritis. Current Insights,” Frontiers in Medicine 8 (2021): 782150. 34859023 10.3389/fmed.2021.782150 PMC8630576

[6] S. S. Zhao , S. Robertson , T. Reich , N. L. Harrison , R. J. Moots , and N. J. Goodson , “Prevalence and Impact of Comorbidities in Axial Spondyloarthritis: Systematic Review and Meta‐Analysis,” supplement, Rheumatology 59, no. Suppl4 (2020): iv47–iv57. 33053193 10.1093/rheumatology/keaa246 PMC7566561

[7] J. Rueda‐Gotor , I. Ferraz‐Amaro , F. Genre , et al., “Cardiovascular and Disease‐Related Features Associated With Extra‐Articular Manifestations in Axial Spondyloarthritis. A Multicenter Study of 888 Patients,” Seminars in Arthritis and Rheumatism 57 (2022): 152096. 36150319 10.1016/j.semarthrit.2022.152096

[8] A. K. Rausch Osthoff , K. Niedermann , J. Braun , et al., “2018 EULAR Recommendations for Physical Activity in People With Inflammatory Arthritis and Osteoarthritis,” Annals of the Rheumatic Diseases 77, no. 9 (2018): 1251–1260. 29997112 10.1136/annrheumdis-2018-213585

[9] D. van der Heijde , S. Ramiro , R. Landewé , et al., “2016 Update of the ASAS‐EULAR Management Recommendations for Axial Spondyloarthritis,” Annals of the Rheumatic Diseases 76, no. 6 (2017): 978–991. 28087505 10.1136/annrheumdis-2016-210770

[10] C. Martey and R. Sengupta , “Physical Therapy in Axial Spondyloarthritis: Guidelines, Evidence and Clinical Practice,” Current Opinion in Rheumatology 32, no. 4 (2020): 365–370. 32453037 10.1097/BOR.0000000000000714

[11] S. H. Sveaas , I. J. Berg , S. A. Provan , et al., “Efficacy of High Intensity Exercise on Disease Activity and Cardiovascular Risk in Active Axial Spondyloarthritis: A Randomized Controlled Pilot Study,” PLoS One 9, no. 9 (2014): e108688. 25268365 10.1371/journal.pone.0108688 PMC4182541

[12] S. H. Sveaas , A. Bilberg , I. J. Berg , et al., “High Intensity Exercise for 3 Months Reduces Disease Activity in Axial Spondyloarthritis (axSpA): A Multicentre Randomised Trial of 100 Patients,” British Journal of Sports Medicine 54, no. 5 (2020): 292–297. 30745314 10.1136/bjsports-2018-099943

[13] M. J. Gibala , J. P. Little , M. J. Macdonald , and J. A. Hawley , “Physiological Adaptations to Low‐Volume, High‐Intensity Interval Training in Health and Disease,” Journal of Physiology 590, no. 5 (2012): 1077–1084. 22289907 10.1113/jphysiol.2011.224725 PMC3381816

[14] J. Gallo‐Villegas , J. C. Aristizabal , M. Estrada , et al., “Efficacy of High‐Intensity, Low‐Volume Interval Training Compared to Continuous Aerobic Training on Insulin Resistance, Skeletal Muscle Structure and Function in Adults With Metabolic Syndrome: Study Protocol for a Randomized Controlled Clinical Trial (Intraining‐MET),” Trials 19, no. 1 (2018): 144. 29482601 10.1186/s13063-018-2541-7 PMC5828481

[15] J. M. Leiva‐Valderrama , A. Montes‐de‐Oca‐Garcia , E. Opazo‐Diaz , et al., “Effects of High‐Intensity Interval Training on Inflammatory Biomarkers in Patients With Type 2 Diabetes. A Systematic Review,” International Journal of Environmental Research and Public Health 18, no. 23 (2021): 12644. 34886369 10.3390/ijerph182312644 PMC8656922

[16] D. Reljic , W. Dieterich , H. J. Herrmann , M. F. Neurath , and Y. Zopf , “‘HIIT the Inflammation’: Comparative Effects of Low‐Volume Interval Training and Resistance Exercises on Inflammatory Indices in Obese Metabolic Syndrome Patients Undergoing Caloric Restriction,” Nutrients 14, no. 10 (2022): 1996. 35631137 10.3390/nu14101996 PMC9145085

[17] F. B. Benatti and B. K. Pedersen , “Exercise as an Anti‐Inflammatory Therapy for Rheumatic Diseases‐Myokine Regulation,” Nature Reviews Rheumatology 11, no. 2 (2015): 86–97. 25422002 10.1038/nrrheum.2014.193

[18] M. L. Bay and B. K. Pedersen , “Muscle‐Organ Crosstalk: Focus on Immunometabolism,” Frontiers in Physiology 11 (2020): 567881. 33013484 10.3389/fphys.2020.567881 PMC7509178

[19] A. Steensberg , C. P. Fischer , C. Keller , K. Møller , and B. K. Pedersen , “IL‐6 Enhances Plasma IL‐1ra, IL‐10, and Cortisol in Humans,” American Journal of Physiology‐Endocrinology and Metabolism 285, no. 2 (2003): E433–E437. 12857678 10.1152/ajpendo.00074.2003

[20] P. Libby and G. K. Hansson , “From Focal Lipid Storage to Systemic Inflammation,” Journal of the American College of Cardiology 74, no. 12 (2019): 1594–1607. 31537270 10.1016/j.jacc.2019.07.061 PMC6910128

[21] P. Kowiański , G. Lietzau , E. Czuba , M. Waśkow , A. Steliga , and J. Moryś , “BDNF: A Key Factor With Multipotent Impact on Brain Signaling and Synaptic Plasticity,” Cellular and Molecular Neurobiology 38, no. 3 (2018): 579–593. 28623429 10.1007/s10571-017-0510-4 PMC5835061

[22] I. M. Olfert , O. Baum , Y. Hellsten , and S. Egginton , “Advances and Challenges in Skeletal Muscle Angiogenesis,” American Journal of Physiology‐Heart and Circulatory Physiology 310, no. 3 (2016): H326–H336. 26608338 10.1152/ajpheart.00635.2015 PMC4796623

[23] X. Baraliakos , M. Østergard , R. G. Lambert , et al., “MRI Lesions of the Spine in Patients With Axial Spondyloarthritis: An Update of Lesion Definitions and Validation by the ASAS MRI Working Group,” Annals of the Rheumatic Diseases 81, no. 9 (2022): 1243–1251. 35609977 10.1136/annrheumdis-2021-222081

[24] G. P. Dorneles , D. O. Haddad , V. O. Fagundes , et al., “High Intensity Interval Exercise Decreases IL‐8 and Enhances the Immunomodulatory Cytokine interleukin‐10 in Lean and Overweight‐Obese Individuals,” Cytokine 77 (2016): 1–9. 26476404 10.1016/j.cyto.2015.10.003

[25] B. Rohnejad and A. Monazzami , “Effects of High‐Intensity Intermittent Training on Some Inflammatory and Muscle Damage Indices in Overweight Middle‐Aged Men,” Apunts Sports Medicine 58, no. 100404 (2023): 100404.

[26] F. Kaspar , H. F. Jelinek , S. Perkins , H. A. Al‐Aubaidy , B. deJong , and E. Butkowski , “Acute‐Phase Inflammatory Response to Single‐Bout HIIT and Endurance Training: A Comparative Study,” Mediators of Inflammation 2016 (2016): 1–6. 10.1155/2016/5474837 PMC4861798 27212809

[27] S. Proschinger , A. Schenk , I. Weßels , et al., “Intensity‐ and Time‐Matched Acute Interval and Continuous Endurance Exercise Similarly Induce an Anti‐Inflammatory Environment in Recreationally Active Runners: Focus on PD‐1 Expression In T(REGS) and the IL‐6/IL‐10 Axis,” European Journal of Applied Physiology 123, no. 11 (2023): 2575–2584. 37336816 10.1007/s00421-023-05251-y PMC10615943

[28] M. Rudwaleit , D. van der Heijde , R. Landewé , et al., “The Development of Assessment of Spondyloarthritis International Society Classification Criteria for Axial Spondyloarthritis (Part II): Validation and Final Selection,” Annals of the Rheumatic Diseases 68, no. 6 (2009): 777–783. 19297344 10.1136/ard.2009.108233

[29] P. O. Åstrand , T. E. Cuddy , B. Saltin , and J. Stenberg , “Cardiac Output During Submaximal and Maximal Work,” Journal of Applied Physiology 19 (1964): 268–274. 14155294 10.1152/jappl.1964.19.2.268

[30] G. Borg Borg's Perceived Exertion and Pain Scales: Human Kinetics; 1998.

[31] J. L. Taylor , D. J. Holland , J. G. Spathis , et al., “Guidelines for the Delivery and Monitoring of High Intensity Interval Training in Clinical Populations,” Progress in Cardiovascular Diseases 62, no. 2 (2019): 140–146. 30685470 10.1016/j.pcad.2019.01.004

[32] S. Garrett , T. Jenkinson , L. G. Kennedy , H. Whitelock , P. Gaisford , and A. Calin , “A New Approach to Defining Disease Status in Ankylosing Spondylitis: The Bath Ankylosing Spondylitis Disease Activity Index,” Journal of Rheumatology 21, no. 12 (1994): 2286–2291. 7699630

[33] G. EuroQol , “EuroQol‐A New Facility for the Measurement of Health‐Related Quality of Life,” Health Policy 16, no. 3 (1990): 199–208. 10109801 10.1016/0168-8510(90)90421-9

[34] J. B. Gillen , B. J. Martin , M. J. MacInnis , L. E. Skelly , M. A. Tarnopolsky , and M. J. Gibala , “Twelve Weeks of Sprint Interval Training Improves Indices of Cardiometabolic Health Similar to Traditional Endurance Training Despite a Five‐Fold Lower Exercise Volume and Time Commitment,” PLoS One 11, no. 4 (2016): e0154075. 27115137 10.1371/journal.pone.0154075 PMC4846072

[35] J. S. Ramos , L. C. Dalleck , A. E. Tjonna , K. S. Beetham , and J. S. Coombes , “The Impact of High‐Intensity Interval Training Versus Moderate‐Intensity Continuous Training on Vascular Function: A Systematic Review and Meta‐Analysis,” Sports Medicine 45, no. 5 (2015): 679–692. 25771785 10.1007/s40279-015-0321-z

[36] J. J. Edwards , M. Griffiths , A. H. P. Deenmamode , and J. M. O'Driscoll , “High‐Intensity Interval Training and Cardiometabolic Health in the General Population: A Systematic Review and Meta‐Analysis of Randomised Controlled Trials,” Sports Medicine 53 (2023): 1753–1763. 37204620 10.1007/s40279-023-01863-8

[37] R. Mateo‐Gallego , L. Madinaveitia‐Nisarre , J. Giné‐Gonzalez , et al., “The Effects of High‐Intensity Interval Training on Glucose Metabolism, Cardiorespiratory Fitness and Weight Control in Subjects With Diabetes: Systematic Review a Meta‐Analysis,” Diabetes Research and Clinical Practice 190 (2022): 109979. 35780905 10.1016/j.diabres.2022.109979

[38] R. Martland , V. Mondelli , F. Gaughran , and B. Stubbs , “Can High‐Intensity Interval Training Improve Physical and Mental Health Outcomes? A Meta‐Review of 33 Systematic Reviews Across the Lifespan,” Journal of Sports Sciences 38, no. 4 (2020): 430–469. 31889469 10.1080/02640414.2019.1706829

[39] L. Croft , J. D. Bartlett , D. P. M. MacLaren , et al., “High‐Intensity Interval Training Attenuates the Exercise‐Induced Increase in Plasma IL‐6 in Response to Acute Exercise,” Applied Physiology, Nutrition, and Metabolism 34, no. 6 (2009): 1098–1107. 10.1139/H09-117 20029520

[40] K. Zwetsloot , C. John , M. Lawrence , R. Battista , and R. A. Shanely , “High‐Intensity Interval Training Induces a Modest Systemic Inflammatory Response in Active, Young Men,” Journal of Inflammation Research 7 (2014): 9–17. 24520199 10.2147/JIR.S54721 PMC3920540

[41] C. Aluganti Narasimhulu and D. K. Singla , “The Role of Bone Morphogenetic Protein 7 (BMP‐7) in Inflammation in Heart Diseases,” Cells 9, no. 2 (2020): 280. 31979268 10.3390/cells9020280 PMC7073173

[42] E. Descamps , A. Molto , D. Borderie , et al., “Changes in Bone Formation Regulator Biomarkers in Early Axial Spondyloarthritis,” Rheumatology 60, no. 3 (2021): 1185–1194. 32888036 10.1093/rheumatology/keaa296

[43] D. K. Singla , R. Singla , and J. Wang , “BMP‐7 Treatment Increases M2 Macrophage Differentiation and Reduces Inflammation and Plaque Formation in Apo E‐/‐ Mice,” PLoS One 11, no. 1 (2016): e0147897. 26824441 10.1371/journal.pone.0147897 PMC4732822

[44] J. H. Kwon , K. M. Moon , and K. W. Min , “Exercise‐Induced Myokines Can Explain the Importance of Physical Activity in the Elderly: An Overview,” Healthcare 8, no. 4 (2020): 378. 33019579 10.3390/healthcare8040378 PMC7712334

[45] A. Mahmoud , D. Fayez , M. M. Abou Gabal , S. M. Hosny Hamza , and T. Badr , “Insight on Bone Morphogenetic Protein 7 in Ankylosing Spondylitis and Its Association With Disease Activity and Radiographic Damage,” Electronic Physician 8, no. 7 (2016): 2670–2678. 27648196 10.19082/2670 PMC5014508

[46] M. C. Park , Y. B. Park , and S. K. Lee , “Relationship of Bone Morphogenetic Proteins to Disease Activity and Radiographic Damage in Patients With Ankylosing Spondylitis,” Scandinavian Journal of Rheumatology 37, no. 3 (2008): 200–204. 18465455 10.1080/03009740701774941

[47] H. A. Chen , C. H. Chen , Y. J. Lin , et al., “Association of Bone Morphogenetic Proteins With Spinal Fusion in Ankylosing Spondylitis,” Journal of Rheumatology 37, no. 10 (2010): 2126–2132. 20682677 10.3899/jrheum.100200

[48] B. Kliszczewicz , C. D. Markert , E. Bechke , et al., “Acute Effect of Popular High‐Intensity Functional Training Exercise on Physiologic Markers of Growth,” Journal of Strength and Conditioning Research 35, no. 6 (2021): 1677–1684. 30399116 10.1519/JSC.0000000000002933

[49] G. Manferdelli , N. Freitag , K. Doma , et al., “Acute Hormonal Responses to High‐Intensity Interval Training in Hyperoxia,” Journal of Human Kinetics 73 (2020): 125–134. 32774544 10.2478/hukin-2019-0137 PMC7386136

[50] H. Appel , L. Janssen , J. Listing , R. Heydrich , M. Rudwaleit , and J. Sieper , “Serum Levels of Biomarkers of Bone and Cartilage Destruction and New Bone Formation in Different Cohorts of Patients With Axial Spondyloarthritis With and Without Tumor Necrosis Factor‐Alpha Blocker Treatment,” Arthritis Research and Therapy 10, no. 5 (2008): R125. 18945353 10.1186/ar2537 PMC2592815

[51] S. J. Pedersen , M. L. Hetland , I. J. Sørensen , M. Østergaard , H. J. Nielsen , and J. S. Johansen , “Circulating Levels of interleukin‐6, Vascular Endothelial Growth Factor, YKL‐40, Matrix metalloproteinase‐3, and Total Aggrecan in Spondyloarthritis Patients During 3 Years of Treatment With TNFα Inhibitors,” Clinical Rheumatology 29, no. 11 (2010): 1301–1309. 20640910 10.1007/s10067-010-1528-x

[52] M. Khalafi and M. E. Symonds , “The Impact of High‐Intensity Interval Training on Inflammatory Markers in Metabolic Disorders: A Meta‐Analysis,” Scandinavian Journal of Medicine and Science in Sports 30, no. 11 (2020): 2020–2036. 32585734 10.1111/sms.13754

