# Supplementary material for: Effects on Serum Protein Levels From One Bout of High Intensity Interval Training in Individuals With Axial Spondyloarthritis and Controls
Source: Immun Inflamm Dis. 2025 Dec 14;13(12):e70305. doi: 10.1002/iid3.70305 (PMC12702689; doi:10.1002/iid3.70305)
Supplement: Supplementary file 1 — Supplementary Table 1: Fold change of IL‐6 in serum of healthy individuals (age 18–29) from baseline and from 5 min post HIIT on a group level. [file IID3-13-e70305-s001.docx]

**Supplementary Table 1**

**Supplementary Table 1.** Fold change of IL-6 in serum of healthy individuals

(age 18-29) from baseline and from 5 min post HIIT on a group level.

|  | **Fold change (95% CI)** | |  |  |
| --- | --- | --- | --- | --- |
|  | **5 min post HIIT vs. Baseline** | **1h post HIIT vs.**  **Baseline** | | **1h post HIIT vs.**  **5 min post HIIT** |
| **IL-6** | 2.44 (1.18–5.04)  p= 0.010* | 1.63 (1.47–1.81)  p <0.001** | | 0.67 (0.32–1.36)  p= 0.52 |

Results are presented as fold changes from baseline and from 5 min post HIIT

with 95% CI for measurements 5 minutes and 1h after the HIIT-session. *p<0.05

based on repeated measures ANOVA on log-transformed variable. **p<0.01.

n=12.
